# Veterinary College Notes

**Published:** 1890-01

**Authors:** 


### VETERINARY COLLEGE NOTES.

*Faculty of Comparative Medicine and Veterinary Science.*—McGill University—formerly Montreal Veterinary College. Dr. Johnston has been appointed Demonstrator of Pathological Anatomy and Curator of the Museum. Very important additions have been made to the dissecting room. A new pathological laboratory, furnished with microscopic and other requisites, has been added. The operating yard has been roofed in and the whole establishment has been heated by hot water. Professor Wesley Mills published in October last a valuable text-book on Physiology, Human and Comparative. (Publishers, Appleton & Co.) The entire teaching staff have been appointed Professors of the Faculty of Comparative Medicine and Veterinary Science of the McGill University, the Principal, D. McEachean, being appointed Dean of the Faculty. The Dean is chief inspector of stock and cattle quarantines for Canada, Professors Baker and C. McEachean being inspectors. He is also Veterinary advisor to the Provincial Government, and a Justice of the Peace with jurisdiction over the entire Province of Quebec. Valuable scientific investigations and publications of Pathological reports have been made by A.W. Clement, Baltimore, and reports on maladie de coit by W. Williams of Bloomington Ills., a graduate of this college, also reflects to its credit. There have been appointed as teachers of Veterinary Science, in Chicago Veterinary College, Minneapolis Veterinary School, Columbus, Mo., Agricnltural College, State Agricultural College, Fort Collins, Col., Austin Baker, G. G. Lyford, Paul Paquin and Wm. McEachean, M.D.V.S., in the United States, and J. A. Couture in the French Veterinary School at Quebec, uphold the reputation of their alma mater. The latter has published a valuable work on Dairy Cattle. V. T. Daubigny is in the Veterinary Department of Luval University, (French) at Montreal, in Canada.

*North Western Veterinary College.*—The course of instruction has been discontinued for the present season, owing to a disastrous fire on December 1, which destroyed the buildlngs, museum, and a number of horses, including Professor Lyford's valuable stallions Col. West and Blackmont. In addition to the direct pecuniary loss, which is very great, the loss of material is irreparable, and the misfortune to the faculty and inconvenience to the students will be seriously regretted by the profession throughout the country. Dr. J. J. Bradley has been assisting Dr. Halloway, Territorial Veterinarian of Montana.

*Chicago Veterinary College.*—A pathological laboratory has been established under the direction of Dr. F. S. Billings. Dr. Chas. E. Sayer, D.V,S., has been appointed to a chair of Veterinary Dentistry and Oral Surgery, and these two courses have been added to the curriculum. An additional story has been added to the building, more than doubling its capacity, in lecture rooms, dissecting room, chemical laboratory, museum and hospital. The enlarged building can accommodate comfortably 250 students.

